# An Abundant Tissue Macrophage Population in the Adult Murine Heart with a Distinct Alternatively-Activated Macrophage Profile

**DOI:** 10.1371/journal.pone.0036814

**Published:** 2012-05-10

**Authors:** Alexander R. Pinto, Rosa Paolicelli, Ekaterina Salimova, Janko Gospocic, Esfir Slonimsky, Daniel Bilbao-Cortes, James W. Godwin, Nadia A. Rosenthal

**Affiliations:** 1 European Molecular Biology Laboratory (EMBL) Mouse Biology Unit, Monterotondo (Rome), Italy; 2 Australian Regenerative Medicine Institute (ARMI), Monash University, Clayton, Victoria, Australia; 3 Harefield Heart Science Centre, National Heart and Lung Institute, Imperial College London, London, United Kingdom; University of Chicago, United States of America

## Abstract

Cardiac tissue macrophages (cTMs) are a previously uncharacterised cell type that we have identified and characterise here as an abundant GFP^+^ population within the adult *Cx_3_cr1^GFP/+^* knock-in mouse heart. They comprise the predominant myeloid cell population in the myocardium, and are found throughout myocardial interstitial spaces interacting directly with capillary endothelial cells and cardiomyocytes. Flow cytometry-based immunophenotyping shows that cTMs exhibit canonical macrophage markers. Gene expression analysis shows that cTMs (CD45^+^CD11b^+^GFP^+^) are distinct from mononuclear CD45^+^CD11b^+^GFP^+^ cells sorted from the spleen and brain of adult *Cx_3_cr1^GFP/+^* mice. Gene expression profiling reveals that cTMs closely resemble alternatively-activated anti-inflammatory M2 macrophages, expressing a number of M2 markers, including Mrc1, CD163, and Lyve-1. While cTMs perform normal tissue macrophage homeostatic functions, they also exhibit a distinct phenotype, involving secretion of salutary factors (including IGF-1) and immune modulation. In summary, the characterisation of cTMs at the cellular and molecular level defines a potentially important role for these cells in cardiac homeostasis.

## Introduction

Macrophages and dendritic cells (DCs) are mononuclear phagocytes (MPs), playing an important role in tissue homeostasis and serve as sentinels for tissue damage and foreign antigens. Tissue MPs, comprised of tissue macrophages (TMs) and DCs, exhibit significant heterogeneity in their phenotype depending on the local environment [1], [2]. As components of the mononuclear phagocytic system, TMs play an important role in inflammation, tissue remodelling and clearing tissue debris by acting as sentinels for foreign antigens and tissue damage. However to date, a systematic analysis of MPs in the mammalian heart has not been undertaken.

The identification of MPs has been significantly aided by the *Cx_3_cr1^GFP/+^* transgenic mouse, where one allele of the Cx_3_cr1 gene, the receptor for the membrane tethered chemokine fractalkine/Cx_3_cl1 expressed specifically in MPs, has been replaced by the gene encoding enhanced green fluorescent protein (GFP) [3]. Expression of GFP within these mice has been used to identify tissue MPs from a wide array of tissues including the central nervous system (microglia) [3], [4], [5], kidney [6], liver [7], skin [8], intestine [9] and blood vessels [10]. Analysis of resident GFP-expressing cells from these tissues has led to key insights regarding MP characteristics in tissue homeostatic conditions and MP responses to tissue damage and invasion by pathogens. In addition, these studies have highlighted the heterogeneity of MPs from various tissues.

Although a number of studies have characterised different tissue MPs, this cell population has not been systematically investigated in myocardial homeostasis and the specific features of these cells in the heart have remained, until now, unexplored. Activated macrophages can generally be categorised based on their functional phenotypes [11], designated M1 for classically-activated and M2 for alternatively-activated. Both *in vitro* and *in vivo* studies have established that M1 macrophages have an inflammatory phenotype coinciding with early-phases of tissue injury, whereas M2 macrophages have an anti-inflammatory, pro-angiogenic and tissue remodelling phenotype coinciding with late-phases of tissue injury [1], [12]. Although this categorisation is over-simplistic, it is useful in characterising MP phenotypes when considering their tissue functions.

Using the *Cx_3_cr1^GFP/+^* transgenic mouse model, we describe an abundant cardiac tissue macrophage (cTM) population within the adult mouse myocardium. Gene expression analysis reveals several defining characteristics of these cells, which closely resemble M2 macrophages in their gene expression signature. The analysis presented here provides new evidence that cTMs participate in many salutary functions in the heart, and may be critical for normal cardiac homeostasis.

## Materials and Methods

### Mice

Adult *Cx_3_cr1^GFP/+^* transgenic mice were a gift from C. Gross (European Molecular Biology Laboratory, Monterotondo, Italy). All mice used were in the C57BL/6 background; they were maintained in a specific pathogen-free(SPF) environment and fed standard mouse diet *ad libitum*. All procedures conducted on mice were approved by the respective animal ethics committees. Mouse procedures conducted are as described in ethics applications MARP/2010/017 and MARP/2011/014 approved by the Monash Animal Research Platform 2 (MARP2) Animal Ethics Committee.

### Preparation of single cell suspensions from different tissues

Prior to isolation of cells from all tissues, except from the peritoneal cavity, mice were perfused with ice-cold Hank's Balanced Saline Solution (HBSS), through the left ventricle to clear blood. For preparing single cell suspensions from hearts, kidneys and lungs, tissues were isolated, placed in HBSS solution on ice before being finely minced with surgical scissors and incubated in collagenase type II (Worthington Laboratories)/HBSS enzyme solution for 30 minutes at 37°C with gentle agitation. Dissociated cells and undigested tissue were centrifuged and resuspended in collagenase-dispase (Roche Applied Science)/HBSS enzyme solution, and incubated for a further 20 min at 37°C with gentle shaking. Cell/tissue suspensions were filtered through 70 µm filters, centrifuged and resuspended in 2% foetal bovine serum (FBS)/PBS until further analysis. Single cell suspensions from adipose were prepared as described above with the omission of the tissue digestion in collagenase-dispase/HBSS enzyme solution. The adipose tissue cell suspensions were centrifuged after the collagenase type II/HBSS solution incubation floating fatty debris was discarded, and cell pellets resuspended in 2% FBS/PBS, before analysis. Splenic single cell suspensions were prepared by mashing spleens against a 70 µm filter in 2% FBS/PBS using a syringe plunger, and the isolated cell suspensions passed through more 70 µm filters. Red blood cell lysis was conducted with splenic cell suspensions using Red Blood Cell Lysis Solution (10 mM KHCO_3_, 150 mM NH_4_CL, 0.1 mM EDTA pH 8.0). Peritoneal cavity cells were isolated by lavage of mouse peritoneal cavities with Dulbecco's Modified Eagle's Medium (DMEM) and centrifugation of the resulting cell suspensions. Mouse brain single cell suspensions were prepared by mincing tissue with a scalpel and incubating them in collagenase-dispase/HBSS solution supplemented with 0.025 U/ml DNaseI (Roche Applied Science) for 30 min at 37°C with gentle agitation. Cell/tissue suspensions were filtered through 70 µm filters and centrifuged (5 min at 4°C, 300 g). Resulting pellets were resuspended in 37% Percoll solution, and separated by gradient centrifugation by placing cell suspension on top of a dense 70% Percoll solution before centrifugation for 25 min at 4°C. Cells accumulated at the interphase of the two solutions were carefully collected and washed in 2% FBS/HBSS before analysis.

### Flow cytometry and antibodies used

The antibodies used for staining of single cell suspensions after FC receptor blocking with CD16/CD32, and for immunofluorescence microscopy are as listed: F4/80 (clone BM8, eBiocience), MHC-II (IA-b) (clone AF6-120.1, BD Pharmingen), CD14 (clone rmC5-3, BD Pharmingen), CD11c (clone HL3, BD Pharmingen), CD86 (clone GL1, BD Pharmingen), NK1.1 (clone PK136, BD Pharmingen), B220 (clone RA3-6B2, BD Pharmingen), CD3ε (clone 145-2C11, BD Pharmingen), LY6C/G (clone RB6-8C5, Biolegend), Mrc1 (CD206), (clone MR5D4, Biolegend), CD45 (clone 30-F11, Biolegend), CD11b (clone M1/70, Biolegend), CD16/CD32 (clone 93, eBioscience), GFP (Chicken Polyclonal (ab13970), Abcam), Lyve-1 (Rabbit Polyclonal (ab14917), Abcam), CD163 (Rabbit Polyclonal (M-96), Santa Cruz Biotechnology). Dextran uptake was detected by flow cytometry in cardiac single cell suspensions after intravenously injecting mice with 50 µg tetramethylrhodamine-labelled 2 MDa dextran (Invitrogen) 20 hours before cell isolation. Cells were analysed by a FACS-Canto cytometer (Becton Dickinson) using FlowJo Software. Fluorescence activated cell sorting (FACS) was conducted using FACS-ARIA cell sorter (Becton Dickinson). All sorted cells were isolated into DMEM supplemented with 10% FBS.

### Quantitative Real Time PCR (qRT-PCR)

Total RNA was isolated from FACS-sorted CX3CR1 positive cells using RNeasy Mini Kit (QIAGEN). DNaseI treatment and cDNA synthesis was performed using QuantiTect Reverse Transcription Kit (QIAGEN). TaqMan gene expression assays (Applied Biosystems) were used for quantification of the mRNA levels of the genes of interest. Probes for TaqMan Gene Expression Assays are as listed: IL6 (Mm00446190_m1), IL10 (Mm00439614_m1), IL1b (Mm01336189_m1), IGF1 (Mm00439560_M1), Lyve1 (Mm00475056_m1), CXCL1 (Mm00433859_m1), CXCL2 (Mm00436450_m1), Chi3l3 (Mm04213363_u1), TIMP2 (Mm00441825_m1), MMP13 (Mm00439491_m1), Stab1 (Mm00460390_m1), Arg1), (Mm00475988_m1), Hmox1 (Mm00516005_m1), Angpt1 (Mm00456503_m1), Rps18 (Mm02601777_g1), Mouse ACTB (actin, beta), Endogenous Control (4352341E). TaqMan assays for *bActin* (VIC) or *Rps18* (FAM) served as endogenous controls. Data was generated with the Applied Biosystems 7500 Real-Time PCR System. Data analysis was performed using the ΔΔC(T) method.

### Immunostaining and confocal microscopic analysis

For preparation of heart tissue for immunostaining, animals were perfused with fresh ice-cold 4% formaldehyde/PBS through the left ventricle and tissue was harvested and incubated in fresh 4% formaldehyde/PBS overnight. For thick section staining, tissue was sectioned using a vibrating blade microtome (Leica Microsystems). Prepared sections were permeabilised with 0.2% Triton X-100 (Sigma)/PBS solutions, before blocking in 1% goat-serum/0.2% Triton X-100/PBS. Standard immunostaining protocols were used for following steps. The primary antibodies used for staining of sections are summarised above. Biotin-conjugated isolectin B4 (Vector Laboratories) was used to stain vasculature. Alexa Fluor conjugated secondary antibodies (Invitrogen) and streptavidin (Invitrogen) were used for fluorescence staining. Fluorescent microscopy images were obtained using Leica SP5 confocal laser scanning microscope (Leica Microsystems) and Nikon C1 confocal laser scanning microscope (Nikon Instruments). Two and three-dimensional images were prepared using Imaris software (Bitplane), with adjustments made to brightness and contrast.

### Microarray analysis

Expression analysis was conducted on FACS sorted CD45^+^CD11b^+^GFP^+^ cells from the mouse heart, spleen and brain. Total RNA samples were isolated from three independent biological replicates using the RNeasy Mini kit (Qiagen), and were analysed with a Bioanalyzer (Agilent Technologies) system for RNA quality and further processed at Genecore (EMBL Heidelberg, Germany).

Microarray experiments were performed on Affymetrix Genechip® HT Mouse Genome 430.2 Array Plate Sets (Agilent Technologies). Data were extracted and normalised using GeneSpring GX 10.0 software (Build 77727; Agilent Technologies and Strand Life Sciences). Probes were filtered to include those where at least 1 of 3 samples had a normalised intensity value between 3.0 and 13.96. Each dataset was derived from three biologically independent replicate samples. In comparative analyses probes were filtered to include those with p≤0.05 (T-test with Bonferroni adjustment). All microarray data files are available in the ArrayExpress database (www.ebi.ac.uk/arrayexpress) under accession number E-MEXP-3347.

### Statistical analysis

For qRT-PCR experiments, student's t-test (two tailed, assuming normal distribution) was applied using Microsoft Excel software where respective genes from various tissues were tested for significance against values determined from the heart. Differences were considered statistically significant where p≤0.05. Values displayed in histograms represent mean ± SEM.

## Results

### GFP^+^ cells within *Cx_3_cr1^GFP/+^* knock-in mouse hearts are myeloid cells

**Figure 1:**
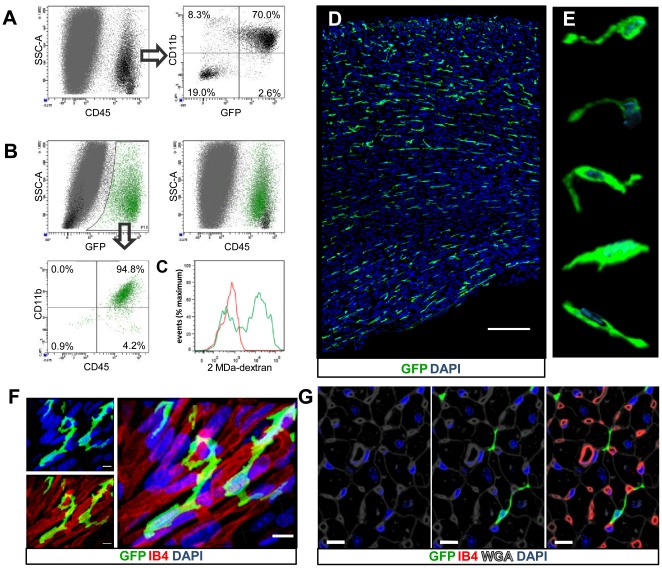
Detection of GFP^+^ cells within adult *Cx_3_cr1^GFP/+^* mouse hearts. (A) Scatter profiles showing CD45^+^ cells in the adult mouse heart (left panel) and the expression of CD11b and GFP within this population (right panel). (B) Scatter profile of GFP^+^ cells (green), with CD45^+^GFP^−^ (black) and CD45^−^GFP^−^ cells and debris (grey) shown (top left panel). (Top right panel) scatter profile of CD45^+^ cells (as shown in figure 1A left panel) with GFP^+^ cells, GFP^−^ cells and debris indicated (green, black and grey respectively; based on gating from figure 1A). (Bottom left panel) scatter profile of CD11b and CD45 expression within GFP^+^ cells. (C) 2 MDa dextran uptake by CD45^+^CD11b^+^GFP^+^ cells. (D) 45 µm projection view of a cross sectional image of the adult *Cx_3_cr1^GFP/+^* mouse heart left ventricle. Scale bar = 150 µm. (E) 3D projections of typical GFP^+^ (green) cells from the left ventricular myocardium with isosurface rendering of nuclei (blue). Figures representative of at least 3 independent experiments. (F) Magnified projection view of GFP^+^ cells with perivascular position shown (capillaries stained with IB4; nuclei stained with DAPI). Scale bar = 5 µm. (G) Optical section of GFP+ cells with perivascular (capillaries stained with IB4) and pericardiomyocyte position (cardiomyocyte cell surface stained with WGA; nuclei stained with DAPI). Scale bar = 10 µm. All images representative of at least 3 similar independent experiments.

To characterise haematopoietic cells, and more precisely myeloid cells, in the adult murine myocardium, we examined GFP^+^ cells extracted from the adult myocardium of *Cx_3_cr1^GFP/+^* knock-in transgenic mice [3]. Flow cytometry analysis of cells isolated from adult *Cx_3_cr1^GFP/+^* mouse hearts identified a large haematopoietic (CD45^+^) and GFP^+^ population (**Fig. 1 a**). Although no clear stratification of GFP^hi^ and GFP^lo^ cells was clearly visible, both GFP^hi^ and GFP^lo^ cells (referred to as GFP^+^) were gated together for subsequent analyses to avoid bias towards over representing data from GFP^hi^ cells. The majority of CD45^+^ cells were GFP^+^, most of which were myeloid as identified by CD11b staining (**Fig. 1 a**). Using an alternative gating approach, we confirmed that GFP^+^ cells from adult *Cx_3_cr1^GFP/+^* mouse hearts were CD45^+^ and CD11b^+^, and that GFP^+^ cells represent a distinct population in scatter profiles of CD45^+^ cells (**Fig. 1 b**). To assess macropinocytosis, a functional hallmark of mononuclear phagocytes [13], we injected *Cx_3_cr1^GFP/+^* mice with TRITC-labelled 2 MDa dextran, which was macropinocytosed by cardiac GFP^+^ cells (**Fig. 1 c**). Confocal fluorescence microscopy of the myocardium revealed stellate shaped GFP^+^ cells distributed throughout the heart (**Fig. 1 d**). These cells are mononuclear and typically have a small cell body with one or two cytoplasmic/dendritic projections (**Fig. 1 e**). The morphology and mononuclear nature of these cells rules out the possibility that they are granulocytes or neutrophils, whereas their extensive macropinocytosis reinforces a cardiac tissue macrophage (cTM) phenotype.

### cTMs are in direct contact with vascular endothelial cells and cardiomyocytes

To determine the anatomical position of cTMs within the adult *Cx_3_cr1^GFP/+^* mouse myocardium in relation to the coronary vasculature, confocal fluorescence microscopy was conducted after isolectin B4 (IB4) staining, which labels endothelial cells. IB4 has previously been shown to stain macrophages [14] and Cx3cr1 expression has been found in endothelial cells of the skin [15]. However, in our experiments we did not observe IB4 staining in cTMs nor did we detect any GFP^+^ endothelial cells. cTMs maintained a perivascular position, associating with at least one capillary endothelial cell (**Fig. 1 f, g**), To determine the position of cTMs in relation to cardiomyocytes, tissue sections were stained with the lectin wheat germ agglutinin (WGA), revealing that cTMs were positioned in direct cell contact with cardiomyocytes (**Fig. 1 g**).

### Immunophenotype of CD45^+^ cells in the hearts of adult *Cx_3_cr1^GFP/+^* knock-in mice

**Figure 2:**
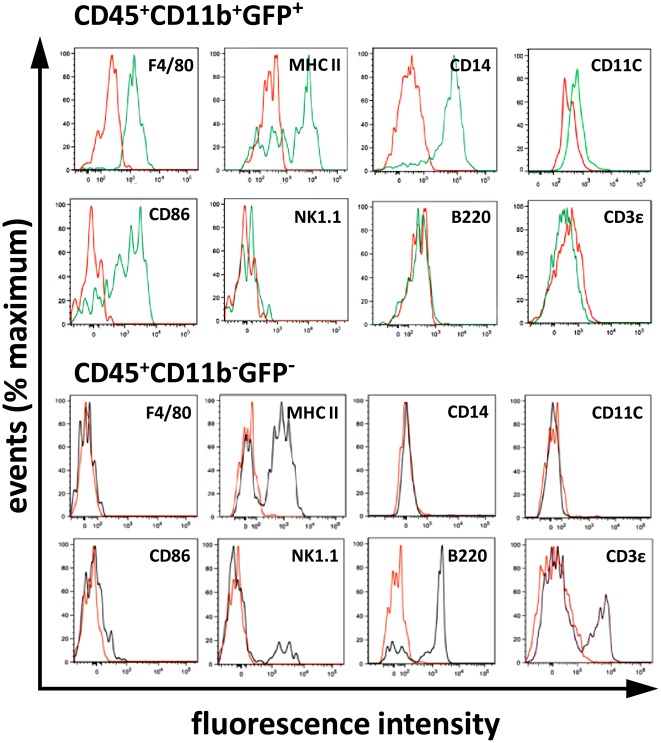
Immunophenotype of cTMs within adult *Cx_3_cr1^GFP/+^* mouse hearts. Representative histograms for myeloid (F4/80, CD11c, MHC-II (IAb), CD14, CD86) and lymphoid (CD3ε, B220) related markers detected in CD45^+^CD11b^+^GFP^+^ population and CD45^+^CD11b^−^GFP^−^ population. Red line shows isotype control staining. Histograms representative of at least 4 experiments, with mouse group sizes of at least 4 mice.

Cell surface marker analysis further distinguished cTMs (CD45^+^CD11b^+^GFP^+^) from the CD45^+^ non-myeloid population (CD45^+^CD11b^−^GFP^−^) (**Fig. 2**). The cTM population stained positive for macrophage marker F4/80, lipo-polysaccharide receptor CD14, and maturation/activation markers MHC-II (IA^b^) and CD86. Low or no staining was detected for CD11c, natural killer cell marker NK 1.1, and lymphocyte markers B220 and CD3ε. These data indicate that cTMs within the adult mouse myocardium are macrophages. In contrast, the cardiac non-myeloid cell population stained for B220 and CD3ε, and a small subset stained positive for NK 1.1, indicating they comprise a lymphoid population and natural killer cells. In addition, these cells stained positive for MHC-II, consistent with the presence of B-cells in this subset, and did not stain for F4/80, CD86, CD14, or CD11c (**Fig. 2**).

### cTMs have a unique gene expression signature compared to GFP^+^ cells in the spleen and brain

**Figure 3:**
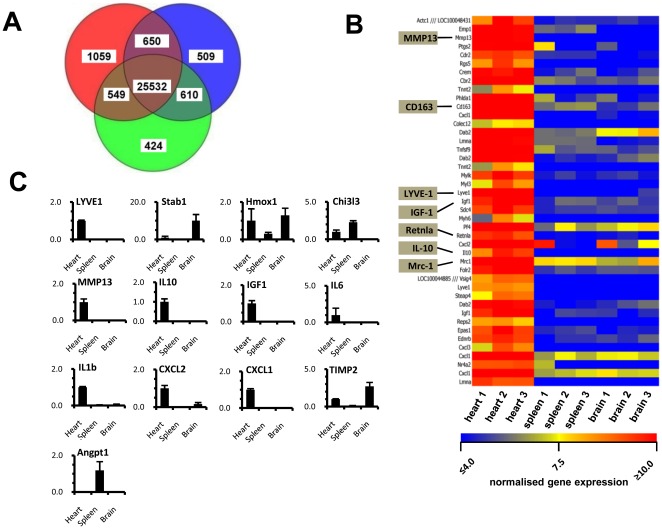
Microarray gene expression analysis of cTMs and GFP^+^ cells from the spleen and brain. (A) Venn-diagram of differentially expressed genes from cTMs (blue) and GFP+ cells from the spleen (green) and brain (red). (B) Heat map of genes enriched greater than 20-fold in cTMs compared to GFP^+^ cells from the spleen and brain. Heat map displays data from each individually isolated GFP^+^ populations. (C) qRT-PCR histograms conducted on selected genes to determine unique enrichment in cTMs (Lyve-1, IGF1, MMP13, IL10, IL6, IL1b, CXCL1, and CXCl2), enrichment in both cTMs and GFP+ cells from the brain (Stab1, Timp2), enrichment in GFP^+^ cells from the spleen (Angpt1) and expression in all three populations (Hmox1). Histograms show mean ± SEM. For microarray analysis, 3 biological replicate samples of GFP^+^ cells from the heart, spleen and brain were prepared in 3 independent isolation procedures for each tissue. Cardiac and brain tissue from 4 mice were used to isolate sufficient number of cells in each independent cell isolation, whereas splenic cells were isolated from at least 2–4 mice.

To develop a gene signature for cTMs, we conducted fluorescent activated cell sorting (FACS) of CD45^+^CD11b^+^GFP^+^ cells from adult *Cx_3_cr1^GFP/+^* mouse hearts (cTMs), spleens and brains and conducted Affymetrix gene array analysis. GFP^+^ cells from adult *Cx_3_cr1^GFP/+^* mouse spleens are comprised of monocytes, macrophages and DCs [16], whereas TMs (microglia) comprise GFP^+^ cells from the brain [17]. Comparison of normalised gene expression between cTMs and GFP^+^ cells from the spleen and brain revealed common features including similar expression levels for housekeeping genes, GAPDH, β-tubulin 1, β-actin and macrophage-specific markers, colony stimulating factor 1 receptor (CSF1-r) and CD68 (**Fig. SI**), confirming the macrophage phenotype of cTMs. Analysis of genes expressed from each cell type (defined as probes expressed above a normalised value of 4) showed 25,532 probes expressed by all three GFP^+^ populations (**Fig. 3 a**). cTMs also expressed 509 unique probes that were not expressed in GFP^+^ cells in the spleen or brain (**Fig. 3 a**). Conversely, splenic GFP^+^ cells expressed 424 unique probes and brain GFP^+^ cells expressed 1059 unique probes (**Fig. 3 a**). Together, these data underscore the heterogeneity of these three myeloid cell populations.

To identify genes that are highly enriched in cTMs, compared to both splenic and brain derived GFP^+^ cells, we filtered probe sets that were 20-fold or more enriched in cTMs (p≤0.05). The filter isolated 42 probes comprising 35 genes (**Fig. 3 b**, **and Table S1**). Of these, at least 12 genes (Lyve-1, Cxcl1, Mmp13, Cxcl2, Pf4, Cd163, Cxcl3, Il10, Tnfsf9, Mrc1, Vsig4, and Colec12) have previously-identified immunological functions. Greater than 100-fold enrichment was observed in cTMs for the genes Lyve-1, Cxcl1, Mmp13, Cxcl2, Cbr1, CD163 and Emp1. Highly expressed cardiomyocyte structural genes were also detected (Mylk, Actc1, Tnnt2, Myl3 and Myh6). Although cardiomyocyte genes were only moderately enriched, the presence of cardiomyocyte transcripts indicates a degree of contamination in cTM RNA samples.

In further comparative analyses, 21 probe sets were identified as 20-fold or greater enriched (p≤0.05) in both cTMs and GFP^+^ cells from the brain, in comparison to GFP^+^ cells from the spleen (data not shown). Notably, these included components of complement pathway, macrophage related genes Stab1 and PMP22 [18], [19]. Conversely, 2 probe sets (Bex6, Apol7c) were identified as enriched 20-fold or more (p≤0.05) in GFP^+^ cells from the spleen compared to GFP^+^ cells from the heart or brain.

**Figure 4:**
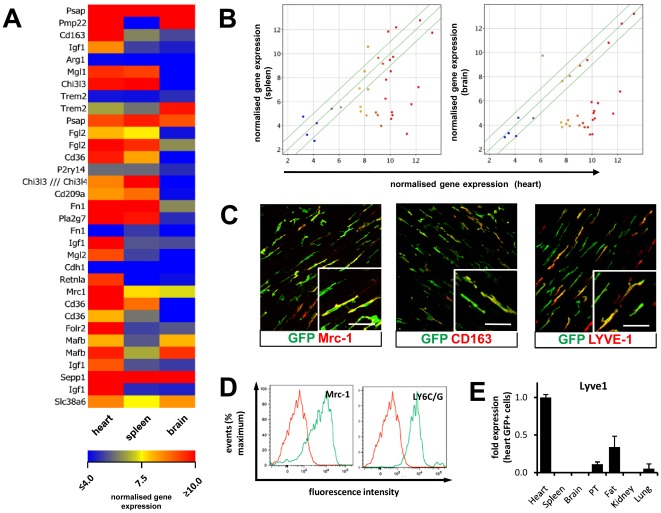
Expression of M2 macrophage markers by cTMs. (A) Heat map of M2 related gene expression by cTMs and GFP^+^ cells from the spleen and brain by microarray analysis. (B) Scatter/correlation profile of M2 related genes expressed by cTMs (heart) and genes expressed in GFP^+^ cells from the spleen and brain. (C) Micrographs of adult *Cx_3_cr1^GFP/+^* mouse heart sections stained for GFP, Mrc1, CD163 and Lyve-1. Scale bar = 30 µm. (D) Flow cytometry histogram showing expression of M2 markers Mrc1 and Ly6C/G by cTMs. Histograms are representative of at least 4 independent experiments. (E) qRT-PCR analysis of Lyve-1 expression by GFP^+^ cells from different tissues. Mean values determined for respective tissues are normalised to means obtained for heart. Histograms show mean ± SEM (n = 3).

Gene expression patterns identified by gene array analysis were validated by quantitative reverse transcriptase-PCR (qRT-PCR), confocal fluorescence microscopy and flow cytometric analysis. For validation of gene expression by qRT-PCR (**Fig. 3 c**), we chose probes enriched in cTMs (Lyve-1, IGF1, MMP13, CXCL1, CXCL2, IL1b, and IL6), enriched in the splenic GFP^+^ cells (Angpt1), or enriched in both cTMs and brain GFP^+^ cells (Timp2, Stab1, Hmox1). qRT-PCR confirmed gene expression patterns observed in microarray analysis, however, levels of enrichment determined by qRT-PCR and gene array analysis varied. Expression of Mrc1, CD163, and Lyve-1 was validated by confocal fluorescence microscopy of adult heart sections from *Cx_3_cr1^GFP/+^* mice (**Fig. 4 c**). In addition, Mrc1 expression was also confirmed by flow cytometric analysis (**Fig. 4 d**).

### cTMs have an M2 macrophage gene expression signature

Microarray gene expression analysis of highly enriched genes in cTMs (Mrc1 and CD163) compared to GFP^+^ cells from the spleen and brain, indicated that these cardiac cells have a distinct gene expression signature resembling alternatively-activated, anti-inflammatory M2 macrophages [11]. To determine if cTMs resemble M2-polarised macrophages, we analysed expression levels of 24 genes reportedly expressed in M2 macrophages *in vitro* and *in vivo* [12], [20]. Indeed, 21 of 24 M2 genes (represented by 34 probe sets) were highly expressed in cTMs (**Fig. 4 a** **and Table S2**). M2 probe sets not expressed or expressed at low levels in cTMs included arginase 1 (Arg1), triggering receptor expressed on myeloid-2 (TREM2), fibronectin 1 (Fn1) and Cadherin 1 (Cdh1). Of these however, one of the two probe sets corresponding to Fn1 was expressed in cTMs at high levels, and one of two probe sets corresponding to Trem2 was expressed at intermediate levels (indicating both genes are expressed in cTMs). Scatter profiles of the M2-related genes examined in the three GFP^+^ populations revealed that cTMs expressed significantly more M2 genes than did splenic and brain GFP^+^ cells (**Fig. 4 b** ****). Although splenic GFP^+^ cells were found to express several M2-genes, brain GFP^+^ cells were found to express very few, indicating a difference in phenotype in the three GFP^+^ populations analysed, with cTMs strongly resembling M2-polarised macrophages. Expression of the canonical M2-macrophage genes, Mrc1 and CD163, was confirmed by confocal fluorescence microscopy (**Fig. 4 c** **and Fig. S2**). Expression of Lyve-1, considered another M2-macrophage related gene [21], [22], and a gene enriched in tumour infiltrating TIE-2 expressing monocytes (TEMs) [23], was also confirmed by confocal fluorescence microscopy (**Fig. 4 c** **and Fig. S2**).

The M2-phenotype of cTMs was further demonstrated by flow cytometry, by determining expression levels of LY6C/G, which is expressed in high levels in M1 and low levels in M2 macrophages [24]. Additionally, we confirmed expression of Mrc1 and low levels of LY6C/G in cTMs, with almost all cells comprising the low expression peak, and a small fraction of cells being present in the high expression peak (**Fig. 4 d**). Consistent with the LY6C/G^lo^ expression, almost all cTMs expressed Mrc1. To determine if cTMs have a distinct gene expression profile amongst GFP^+^ cells from various tissues in *Cx_3_cr1^GFP/+^* mice, we determined expression levels of Lyve-1 from isolated GFP^+^ cells from other tissues (**Fig. 4 e**). Lyve-1 was selected since initial microarray analyses indicated that it may be a cTM-specific marker. Indeed, we found that Lyve-1 was most highly expressed in cTMs (heart) with much lower expression in cells from adipose tissue (fat), peritoneum (PT) and lung.

### cTMs and GFP^+^ cells from the brain express high levels of specific opsonisation components, including components of complement C1q

**Figure 5:**
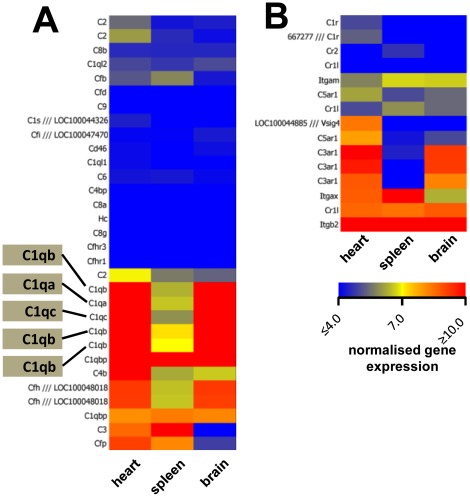
Expression of complement opsonisation components cTMs and GFP^+^ cells from the spleen and brain. Microarray analysis derived heat maps of complement pathway related gene expression by cTMs and GFP^+^ cells from the spleen and brain identified by microarray gene expression analysis. (A) Secreted complement factors with probes corresponding to C1q members highlighted. (B) Complement receptors.

Analysis of common elements between cTMs and brain GFP^+^ (microglia) identified complement components, specifically complement C1q and its subchains (a, b and c) as highly enriched in both cell types, with greater than 20-fold enrichment compared to splenic GFP^+^ cells (**Fig. 5 a** **and Table S3**). Conversely, C3 was found to be highly enriched in splenic GFP^+^ cells and cTMs, whereas all three cell types were found to express C1q binding protein (C1qbp). Variations in complement receptors were also determined by microarray analysis (**Fig. 5 b** **and Table S3**). Complement receptor Vsig4 was uniquely detected in cTMs and complement receptor C3ar1 was highly enriched in cTMs and microglia. Complement receptors Itgam (CD11b), Itgax (CD11c), Itgb2 (CD18) and Cr1l were enriched in all three cell types.

### Gene expression heterogeneity in cTMs and GFP^+^ cells from other tissues

**Figure 6:**
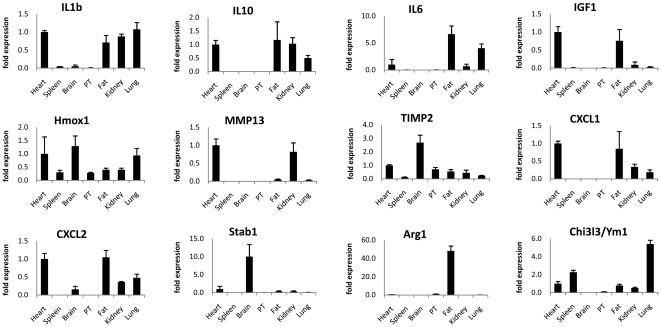
Differential gene expression by GFP^+^ populations from adult *Cx_3_cr1^GFP/+^* mouse tissues determined by qRT-PCR. Means for respective genes from various tissues are normalised to the means obtain for heart (IL1β, IL6, CXCL1, CXCL2, IL10, IGF1, MMP13, Timp2, and Hmox1), lung (Angpt1), or peritoneum (PT; Arg1). Histograms show mean ± SEM (n = 3 independent cell and RNA isolation experiments). RNA for 3 biological replicate samples of GFP^+^ cells of various tissues were prepared in 3 independent isolation procedures for each tissue. For all tissues except spleen, GFP^+^ cells were isolated from 4–7 mice in each independent cell isolation. Splenic GFP^+^ cells were isolated from groups of 2–4 mice per replicate.

To investigate the heterogeneity of gene expression between GFP^+^ cells from different tissues (heart, kidney, lung, adipose (fat), brain, peritoneum, and spleen), qRT-PCR was conducted on a selection of genes including inflammatory and tissue remodelling genes also used for validating microarray data. Both inflammatory and anti-inflammatory cytokines, IL1β and IL10, were expressed in GFP^+^ cells from the heart, kidney, lung and adipose tissue (**Fig. 6**). Chemokines Cxcl1, Cxcl2, and IL6 were also detected in these GFP^+^ populations at different levels. Although macrophages are regarded as a major extrahepatic source of IGF-1, IGF-1 expression was only detected in cTMs and adipose tissue GFP^+^ cells, with low or no transcripts in GFP^+^ cells from other tissues. In addition, matrix metalloprotease 13 (MMP13) was detected in cTMs and GFP^+^ cells from the kidney, with low or no levels in other tissues, whereas Heme oxygenase 1 (Hmox1) and tissue inhibitor of matrix metalloproteases 2 (TIMP2) were detected in all tissues. Thus cTMs have a distinctive gene expression profile compared to TMs from other tissue sources.

## Discussion

This report characterises a novel, abundant population of macrophages within cardiac tissue (cTMs) marked by GFP expression in the *Cx_3_cr1^GFP/+^* knock-in mouse. Immunophenotyping and gene expression analysis confirm that these cells are cardiac tissue macrophages expressing canonical macrophage genes such as F4/80, CD14 and Csf1-r. These findings show that cTMs are distinct from aortic and cardiac valve mononuclear phagocytes, which are reported to exhibit a dendritic cell (DC) cell surface profile [25]. Since migration to local lymph nodes after antigen exposure has not been investigated, we cannot formally exclude the possibility that cTMs represent a DC population [26].

The finding that cTMs occupy an interstitial space, and are closely associated with capillary endothelial cells suggests that cTMs might participate in endocrine signalling through the coronary vasculature. Moreover, the direct contact between cTMs and cardiomyocytes indicates that both cells would be receptive to reciprocal paracrine signalling, pointing to a potentially important role for cTMs in cardiomyocyte homeostasis. Indeed, the finding that cTMs have an M2-like gene expression signature suggests that cTMs perform an immunomodulatory and a tissue salutary function in the heart. The rigorous examination of the M2 phenotype by gene array analysis, flow cytometry and microscopy techniques excludes the possibility that M2 gene expression may be induced as an artefact of the cTM isolation procedure. In addition, of the 24 M2 macrophage markers assessed, the canonical M2-markers Mrc1, Fizz1, Ym1, Mgl1, and Mgl2 [12], [20] were expressed. The absence of Arg1, a canonical marker of mouse M2-macrophages, is understandable considering its role in the production of polyamines that are essential for collagen deposition and fibrosis, which characterise M2 macrophage function following injury [27]. Thus these results indicate that, unlike mouse M2 macrophages involved at later phases of inflammation, cTMs in the undamaged heart have a unique phenotype and function.

Complement secretion and modulation by cTMs points to another mechanism whereby these cells might contribute to myocardial homeostasis. cTM expression of Vsig4, a complement binding protein that negatively regulates T-cell activation [28], indicates that cTMs may suppress T-cell activation within the resting myocardium. Moreover, C1q deposition is important for clearance of apoptotic debris by macrophages *in vitro* and *in vivo* and can dampen inflammation while facilitating macrophage phagocytic activity [29], a function also attributed to other cTM-expressed genes, such as the M2-gene TREM2 [30]. Thus, cTMs may play a significant role in dampening local tissue inflammation. Indeed, loss of complement receptors C1 and C2 results in more severe virally induced myocarditis [31]. Consistently, myocarditis is a common manifestation of systemic lupus erythematosus arising from C1q deficiency.

Gene expression analysis of cTMs indicates that they may share a number of characteristics with adipose tissue macrophages, another M2-like macrophage population [22], [32]. In mouse adipose tissue, deficiency of the cytokines IL4 and IL13, important for M2 macrophage polarisation, results in tissue macrophages presenting a classically activated M1 phenotype [33], coinciding with tissue inflammation [34], [35]. Similarly, IL13 deficiency leads to more severe cardiac inflammation compared to controls, resulting in dilated cardiomyopathy after experimentally induced myocarditis [36]. Thus a loss of M2-phenotype in cTMs is likely to have a similarly detrimental inflammatory effect on the heart. Another similarity identified between adipose tissue macrophages and cTMs is the expression of Lyve-1, which in adipose tissue is implicated in hemangiogenesis [37]. Likewise, cTMs express a number of proangiogenic genes including the proangiogenic M2-genes Retnla/Fizz1 [38] and IGF1 [39], [40]. These data are consistent with the established role of macrophages in angiogenesis [41] and their direct role in capillary anastomosis [42]. However, additional microarray analyses show that VEGF isoforms (a, b and c) are not highly expressed in cTMs (data not shown), indicating that cTMs may participate in angiogenesis by non-canonical VEGF-independent pathways [43]. IGF-1 expression in cTMs and GFP^+^ cells from adipose tissue indicates that these cells are also likely to have a tissue salutary role. IGF-1 exerts protective effects in a number of tissues including heart muscle [44], skeletal muscle [45], [46] and skin [47], and has immunomodulatory potential [44], [46]. However, unlike GFP^+^ cells from adipose tissue, cTMs did not express Arg1, indicating a difference in tissue remodelling function. Consistently we found cTMs expressed the collagenolytic enzyme MMP13, implicated in scar-free liver regeneration [48]. These findings indicate that cTMs may have an antifibrotic role in the heart, distinct from adipose tissue macrophages.

While we have characterised cTMs, with an emphasis on their unique gene expression profile and potential role in cardiac homeostasis, their importance in cardiac homeostasis remains to be confirmed by functional data. Elucidation of their functional role would require cTM-specific ablation strategies, which are currently unavailable. Existing mouse macrophage ablation strategies using clodronate-loaded liposomes [49] and myeloid-specific diphtheria toxin receptor expressing mice (such as CD11b-DTR/GFP mice) [50], [51] are limited since they non-specifically ablate macrophages in many tissues [49], [50], [51]. Therefore, changes determined in cardiac development and homeostasis after ablating cTMs using these strategies are confounded due to systemic depletion of myeloid cell populations. A cTM-specific ablation strategy will help determine the role of cTMs in the induction and progression of cardiac inflammation following injury such as myocardial infarction, which recently has been found to involve a carefully orchestrated influx and efflux of monocytes, macrophages and other inflammatory cells [52].

While this study characterises cTMs in the adult murine heart, further studies are required to chart the appearance of cTMs within the myocardium during cardiac development, although previous reports have used *Cx_3_cr1^GFP/+^* knock-in mice to detect macrophage subsets as early as embryonic days 7.5 to 8.5 of development [53], [54]. The cTM gene expression analysis and comparisons with other adult tissue macrophage populations presented here provides a valuable starting point for the generation of new reporters and cTM-specific drivers that will permit further study of cTMs in cardiac development, homeostasis and injury.

In summary, we have shown that the adult mouse heart hosts an abundant population of tissue macrophages. We have shown that these cells exhibit an M2-macrophage-like phenotype, and have the capacity to secrete salutary factors, clear tissue debris and dampen inflammation, suggesting that they may be important for tissue homeostasis. Future studies will characterise these cells during development, cardiac injury and other pathological states. Notably, the adult mammalian heart retains a number of resident stem cell populations with varying pleuripotency and regenerative capacity, which may be sensitive to a number of cTM secreted factors such as IGF-1, influencing stem cell mobility and differentiation in a wide array of tissue contexts including muscle [55], [56], [57]. Moreover, the quality and behaviour of cTMs in clinically relevant states in the obese, diabetic and elderly context need to be examined. Manipulation of these cells during cardiac repair and regeneration, and in chronic inflammatory states, may represent a novel therapeutic avenue for restoring normal cardiac homeostasis after tissue damage or stress.

## Supporting Information

Figure S1 **Scatter/correlation profile of microarray data.** House-keeping and canonical macrophage genes expressed by cTMs (heart) vs GFP^+^ cells from the brain and spleen (top and bottom panels respectively) are shown in the respective magnified views (right panels).
(TIF)

Figure S2 **Heterogeneity of Mrc1, CD163 and Lyve-1 expression in cTMs.** Histogram of proportion of GFP^+^ cells from adult *Cx_3_cr1^GFP/+^* mouse heart sections that stain for Mrc1, CD163 and Lyve-1. Data obtained from confocal micrographs of stained tissue sections as shown in figure 4C. Histograms show mean ± SEM. For each marker, data was obtained from three independent confocal micrograph fields from the mouse myocardium.
(TIF)

Table S1 **Genes 20-fold or greater enriched in cTMs vs GFP^+^ cells from the spleen and brain.** Acronyms H, B and S denote expression values derived from CD45^+^CD11b^+^GFP^+^ cells from the heart, brain and spleen respectively. HvS denotes a ratio of expression values for CD45^+^CD11b^+^GFP^+^ cells from the heart versus spleen. HvB denotes a similar ratio except CD45^+^CD11b^+^GFP^+^ cells from the heart versus brain.
(TIF)

Table S2 **Expression of M2-related genes in cTMs and GFP^+^ cells from the spleen and brain.** Acronyms are the same as in Table S1.
(TIF)

Table S3 **Expression of complement-related genes in cTMs and GFP^+^ cells from the spleen and brain.** Acronyms are the same as in Table S1 except BvS denotes a ratio of expression values for CD45^+^CD11b^+^GFP^+^ cells from the brain versus spleen.
(TIF)
