# Supplementary figures and images for: An Abundant Tissue Macrophage Population in the Adult Murine Heart with a Distinct Alternatively-Activated Macrophage Profile
Source: PLoS One. 2012 May 10;7(5):e36814. doi: 10.1371/journal.pone.0036814 (PMC3349649; doi:10.1371/journal.pone.0036814)

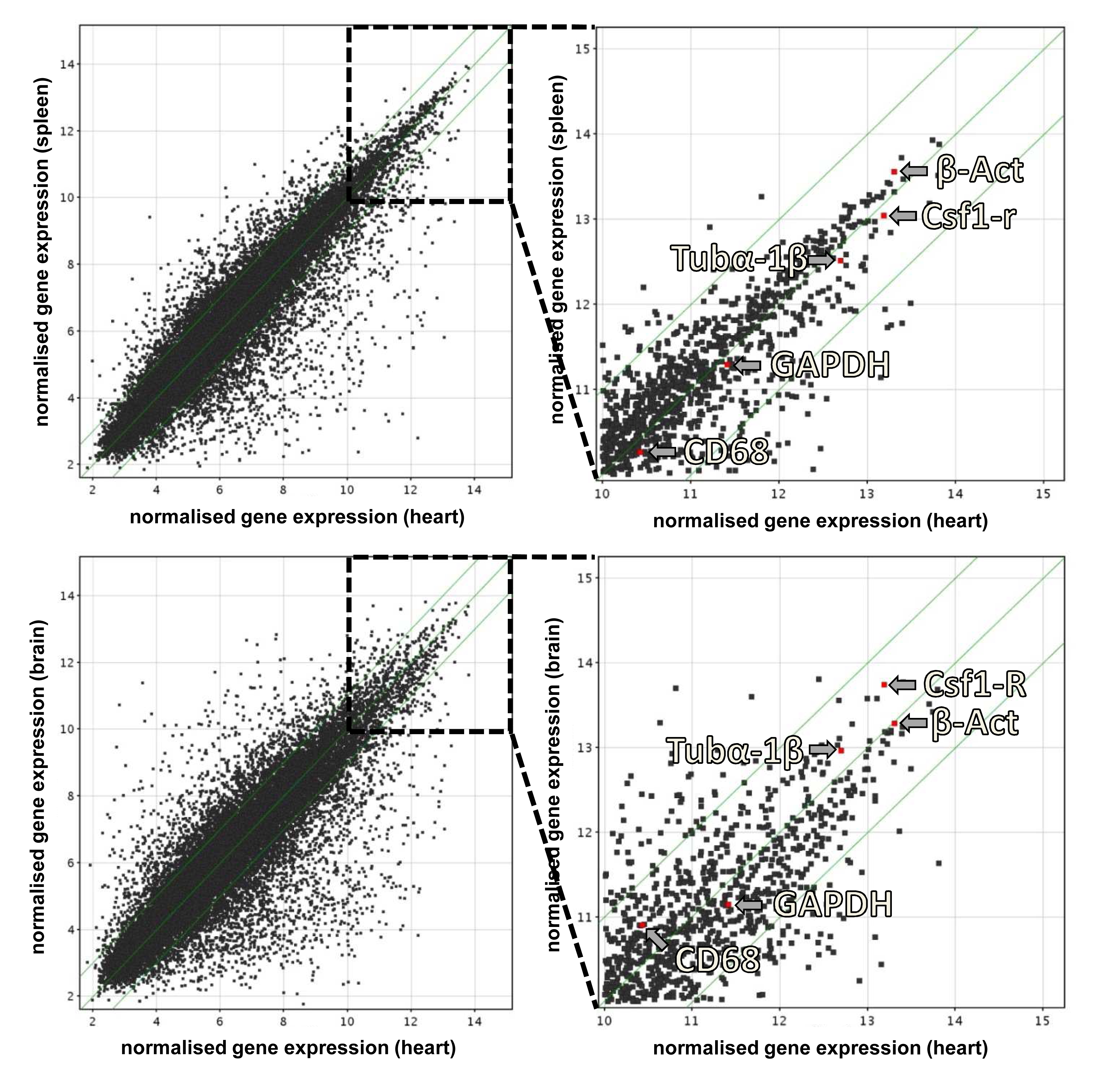

Supplement: Figure S1 — Scatter/correlation profile of microarray data. House-keeping and canonical macrophage genes expressed by cTMs (heart) vs GFP+ cells from the brain and spleen (top and bottom panels respectively) are shown in the respective magnified views (right panels). (TIF) [file pone.0036814.s001.tif]

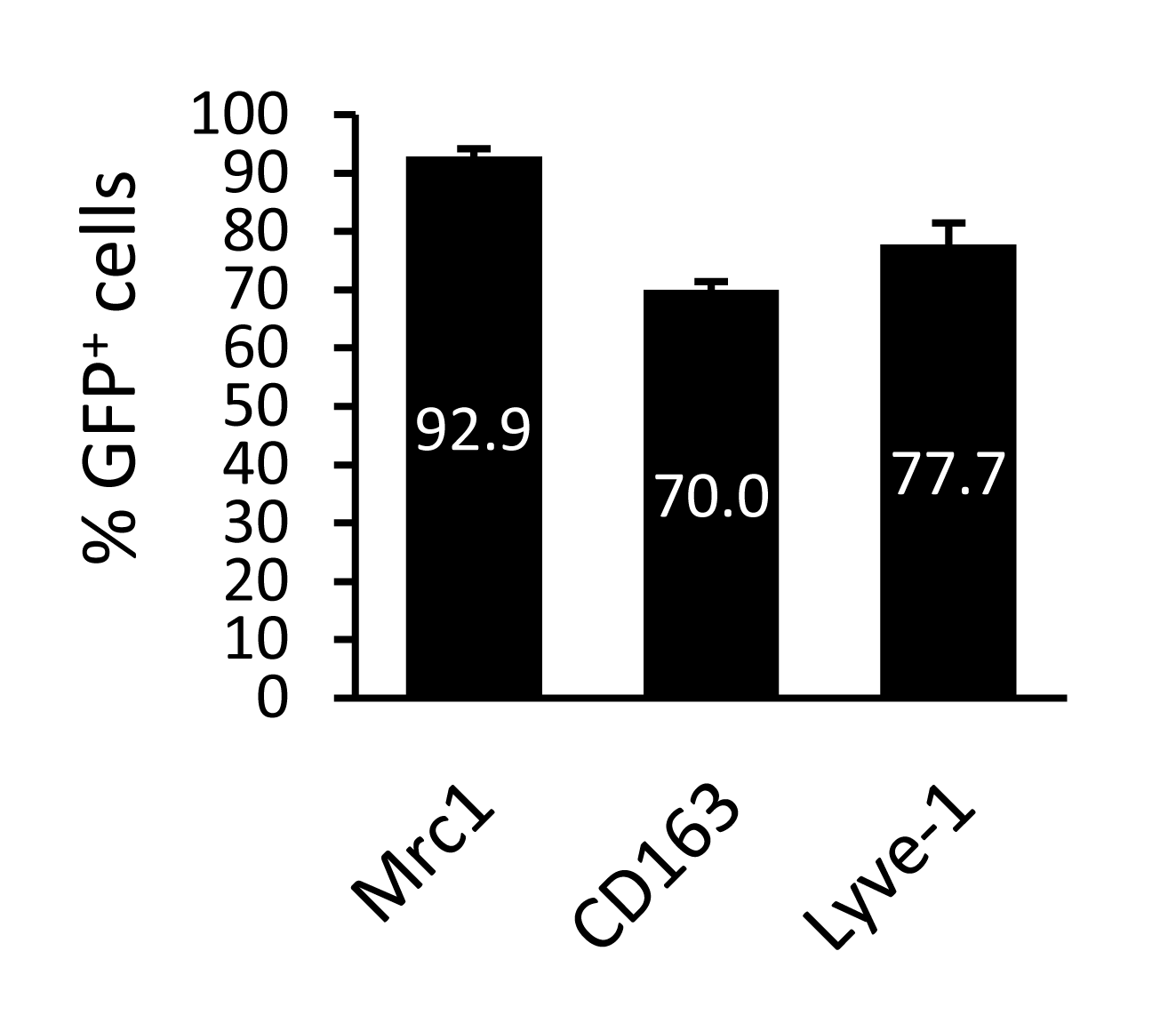

Supplement: Figure S2 — Heterogeneity of Mrc1, CD163 and Lyve-1 expression in cTMs. Histogram of proportion of GFP+ cells from adult Cx3cr1GFP/+ mouse heart sections that stain for Mrc1, CD163 and Lyve-1. Data obtained from confocal micrographs of stained tissue sections as shown in figure 4C. Histograms show mean ± SEM. For each marker, data was obtained from three independent confocal micrograph fields from the mouse myocardium. (TIF) [file pone.0036814.s002.tif]

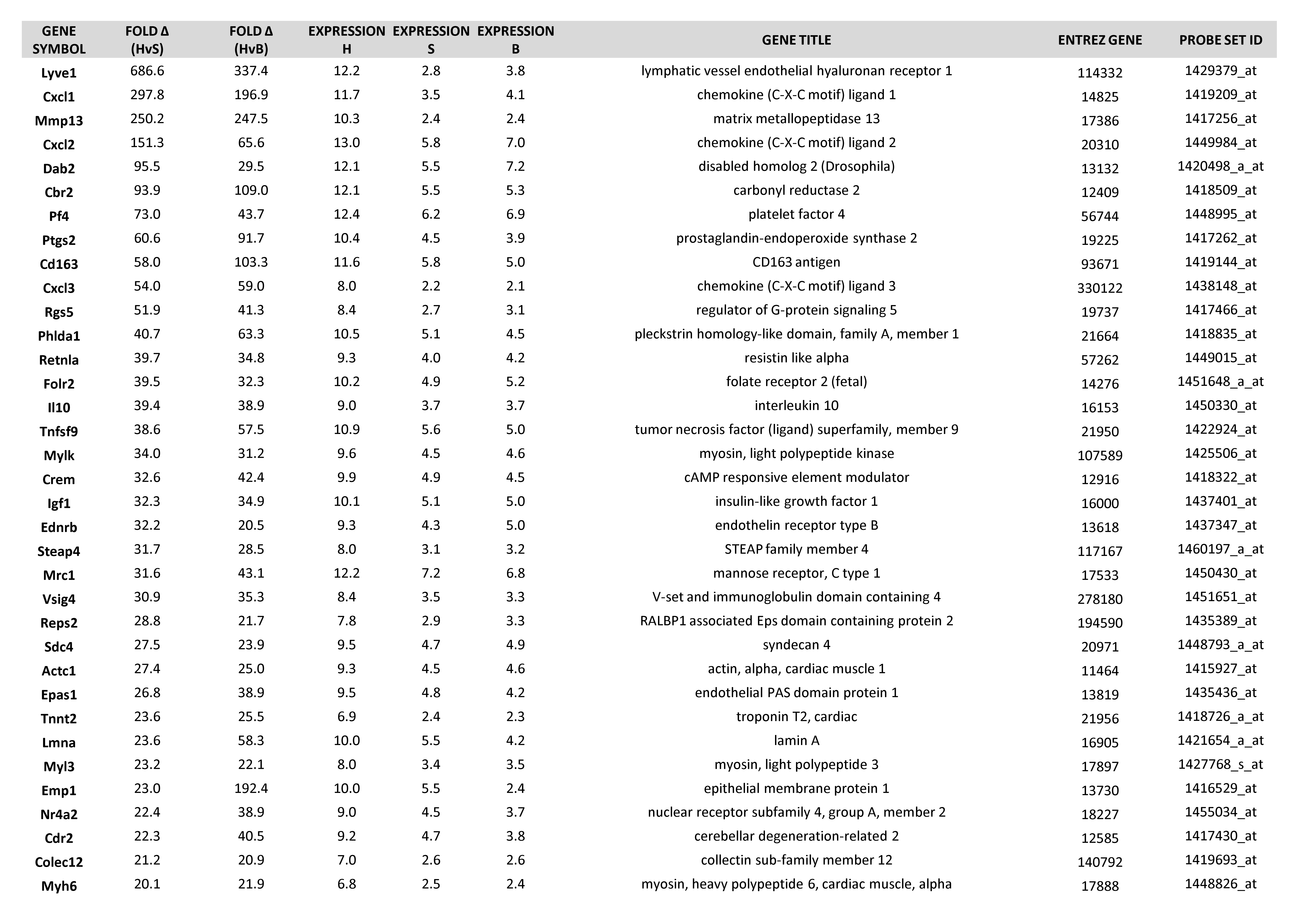

Supplement: Table S1 — Genes 20-fold or greater enriched in cTMs vs GFP+ cells from the spleen and brain. Acronyms H, B and S denote expression values derived from CD45+CD11b+GFP+ cells from the heart, brain and spleen respectively. HvS denotes a ratio of expression values for CD45+CD11b+GFP+ cells from the heart versus spleen. HvB denotes a similar ratio except CD45+CD11b+GFP+ cells from the heart versus brain. (TIF) [file pone.0036814.s003.tif]

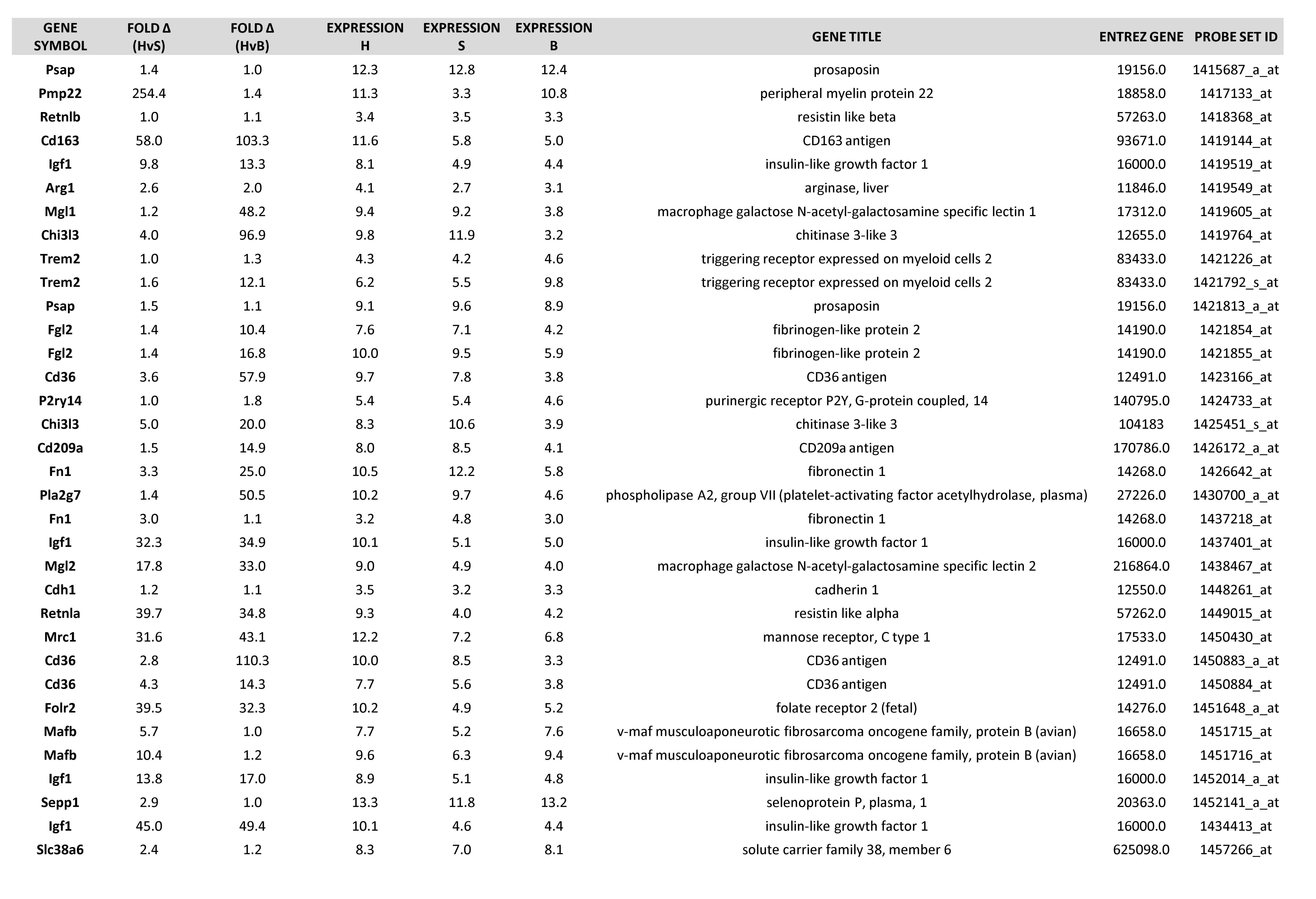

Supplement: Table S2 — Expression of M2-related genes in cTMs and GFP+ cells from the spleen and brain. Acronyms are the same as in Table S1. (TIF) [file pone.0036814.s004.tif]

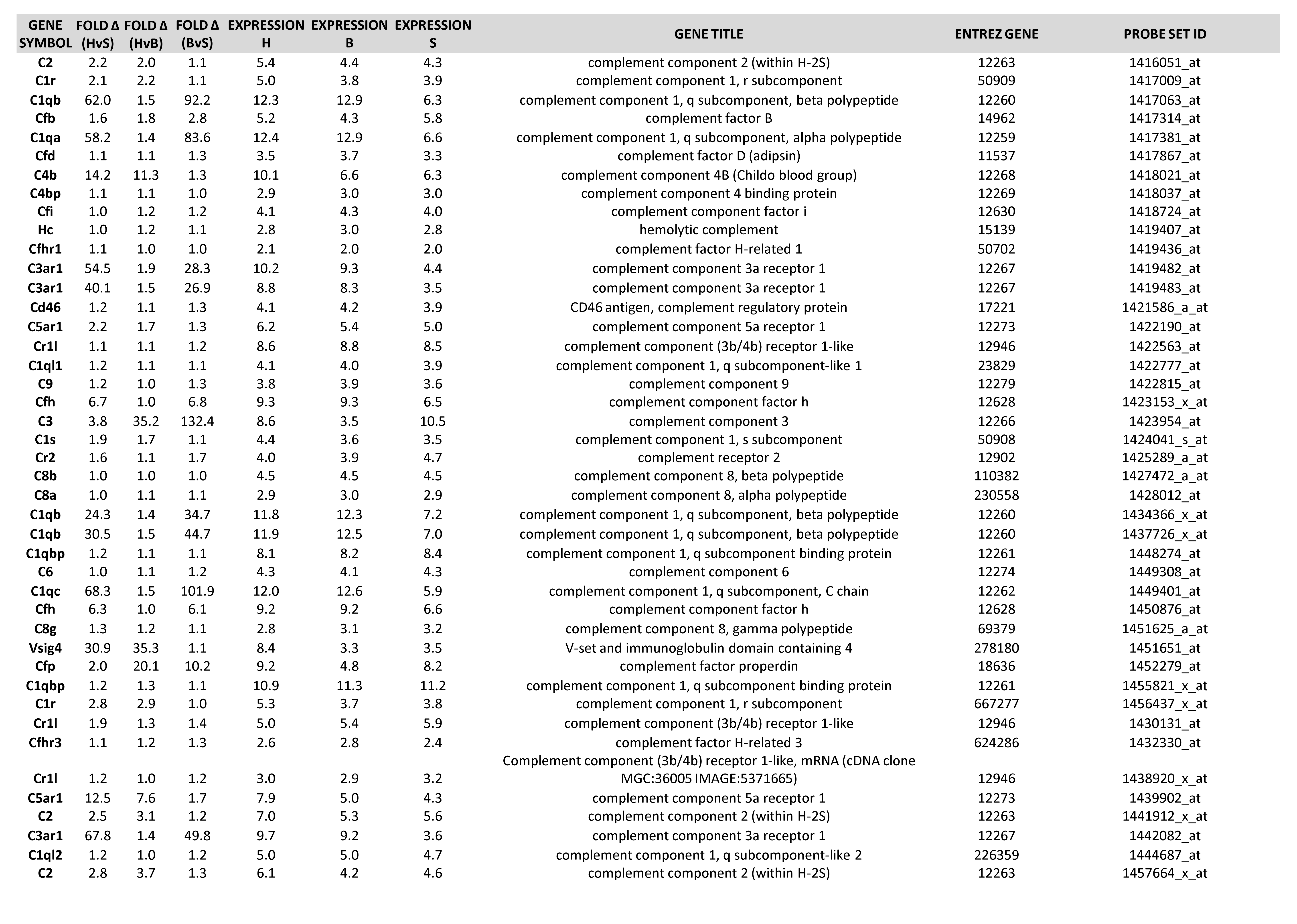

Supplement: Table S3 — Expression of complement-related genes in cTMs and GFP+ cells from the spleen and brain. Acronyms are the same as in Table S1 except BvS denotes a ratio of expression values for CD45+CD11b+GFP+ cells from the brain versus spleen. (TIF) [file pone.0036814.s005.tif]
